# Effect of Freezing Conditions on Fecal Bacterial Composition in Pigs

**DOI:** 10.3390/ani6030018

**Published:** 2016-02-25

**Authors:** Barbara U. Metzler-Zebeli, Peadar G. Lawlor, Elizabeth Magowan, Qendrim Zebeli

**Affiliations:** 1 Department of Farm Animals and Veterinary Public Health, UniversityClinic for Swine, Vetmeduni Vienna, Veterinaerplatz 1, 1210 Vienna, Austria; 2 Pig Development Department, Animal and Grassland Research and Innovation Centre, Teagasc, Moorepark, Fermoy, Co. Cork P61 C996, Ireland; peadar.lawlor@teagasc.ie; 3 Agri-Food and and Biosciences Institute, Large Park, Hillsborough, N. Ireland BT26 6DR, UK; Elizabeth.Magowan@afbini.gov.uk; 4 Department of Farm Animals and Veterinary Public Health, Institute of Animal Nutrition and Functional Plant Compounds, Vetmeduni Vienna, Veterinaerplatz 1, 1210 Vienna, Austria; qendrim.zebeli@vetmeduni.ac.at

**Keywords:** bacterial composition, DNA, feces, freezing condition, pig, quantitative PCR

## Abstract

**Simple Summary:**

Storage of gut samples may affect the extractability of intact DNA and analyzed bacterial composition. In this study, we compared the DNA yield and the abundance of total bacteria and eight bacterial taxa when DNA was extracted from fresh fecal samples of pigs or from freeze stored samples with or without prior snap-freezing in liquid nitrogen. Results showed that the greatest differences in DNA yield and bacterial abundances were found when DNA was extracted from fresh feces compared to freeze stored fecal samples.

**Abstract:**

Sample preservation and recovery of intact DNA from gut samples may affect the inferred gut microbiota composition in pigs. This study aimed to evaluate the effect of the freezing process and storage temperature prior to DNA extraction on DNA recovery and bacterial community composition in pig feces using quantitative PCR. Fresh fecal samples from six growing pigs were collected and five aliquots of each prepared: (1) total DNA extracted immediately; (2) stored at −20 °C; (3) snap frozen and stored at −20 °C; (4) stored at −80 °C; and (5) snap frozen and stored at −80 °C. Results showed that DNA yields from fresh fecal samples were, on average, 25 to 30 ng higher than those from the various stored samples. The DNA extracted from fresh samples had more gene copies of total bacteria and all targeted bacterial groups per gram feces compared to DNA extraction from frozen samples. Data presentation also modified the observed effect of freeze storage; as results for *Lactobacillus* group, *Enterococcus* spp., *Streptococcus* spp., *Clostridium* cluster IV, *Bacteroides-Prevotella-Porphyromonas* and *Enterobacteriaceae* showed the opposite effect when expressed as relative abundance, by being greater in freeze stored feces than in fresh feces. Snap freezing increased the relative proportion of *Clostridium* cluster IV by 24%. In conclusion, the freezing process affected DNA yield and bacterial abundances, whereas snap freezing and storage temperature had only little influence on abundances of bacterial populations in pig feces.

## 1. Introduction

Investigating the composition of gut microbiota composition and correlating findings to specific physiological states has shown the importance of the bacterial community present in the gut as a regulatory factor in health and disease [1,2]. The gut microbiota interact with the animal host, thereby influencing growth-related traits, such as feed intake, growth performance, feed digestibility, fermentation, and behaviour, as well as health-related traits, such as immune competence and immune tolerance in pigs and poultry [2,3]. It is well recognized that molecular-based approaches provide a broader understanding about the diversity and distribution of microbial species in the gut of pigs than culture-based approaches alone [2]. In addition to the application of next-generation sequencing to gain a comprehensive understanding of the bacterial composition and diversity, quantitative PCR (qPCR) approaches are widely used to quantify microbial taxa in gut digesta, feces and gut mucosa scrapings and to monitor pathogen and virulence factor abundance in feces of pigs undergoing treatment [4,5,6]. For all these techniques, it is obligatory that the phylogenetic composition of the extracted DNA reflect the original bacterial community composition [7]. However, sample preservation and recovery of intact DNA from gut samples may bias the inferred microbial community composition [8]. Strong evidence exists that the DNA extraction procedure influences DNA yield and microbial abundances in intestinal digesta samples of livestock animals [9]. Moreover, evidence gained from human stool samples showed that the freezing procedure can affect results of the bacterial community composition as well [7]. In livestock animals, however, little work has been done so far to address if the gut microbial community composition may be affected by snap-freezing or freeze storage temperature. Although similar bacterial families inhabit the gut of humans and pigs, niches are often filled with different species [10]; rendering it difficult to transfer results for sample processing effects on the gut microbiota from humans to pigs. It is common practice to freeze and store porcine gut samples after collection prior to DNA extraction for downstream analysis such as qPCR [4]. If the freezing procedure influences the bacterial composition, this may be disturbing for comparisons within and between separate studies. The objective of the present study was to investigate the hypothesis whether the freezing conditions prior to DNA extraction would affect DNA recovery and bacterial community composition in pig feces using qPCR in order to detect subtle changes in bacterial taxa abundance. 

## 2. Material and Methods

### 2.1. Animals and Fecal Sample Processing 

All procedures involving animal handling and treatment were approved by the institutional ethics committee of the University of Veterinary Medicine and the national authority according to paragraph 26 of the Law for Animal Experiments, Tierversuchsgesetz 2012, BGBl. I Nr. 114/2012 (GZ 68.205/0063-WF/II/3b/2014). Freshly voided grab samples from 6 castrated male pigs ((Landrace × Large White) × Piétrain; average BW 38 ± 6.7 kg (SD); 3–4 months of age) housed individually in metabolism cages (1.20 m × 1.00 m) were collected after 7 day-adaptation to their new environment. Pigs had free access to water and were fed a commercial wheat-barley-soybean meal based grower diet (ME, 13.4 MJ/kg, CP, 16.8% as-fed basis) which was offered three times daily at 08:00 h, 12:00 h and 16:30 h at feed allowances that surpassed pigs’ appetite [11]. After collection, feces were kept on ice during transport to the lab and were processed within 30 min of defecation. Before subsampling (5 aliquots per feces sample), feces were thoroughly homogenized. Subsamples were treated as follows: (1) total DNA extracted immediately; (2) stored at −20 °C; (3) snap frozen and stored at −20 °C; (4) stored at −80 °C; and (5) snap frozen and stored at −80 °C. Treatments 2 to 5 were stored for 3 months before DNA extraction. 

### 2.2. Genomic DNA Isolation

Total genomic DNA was extracted from 300-mg fecal samples using the PowerSoil DNA extraction kit (MoBio Laboratories Inc., Carlsbad, CA, USA) which includes a bead-beating step. To ensure proper lysis of bacteria, a heating step at 70 °C for 10 min was introduced between mixing of the digesta sample with buffer C1 and the bead-beating step as recently described [4]. The DNA was eluted using 100 µL of elution buffer provided by the extraction kit. Total DNA concentration was determined by a Qubit 2.0 Fluorometer (Life Technologies, Carlsbad, CA, USA) using the Qubit dsDNA HS Assay Kit (Life Technologies). Sample volumes were adjusted to achieve similar DNA concentrations across samples for qPCR to avoid an impact of the DNA concentration on the amplification results.

### 2.3. Quantitative PCR

Current primer sets (*i.e.*, *Bacteroides-Prevotella-Porphyromonas*, *Enterobacteriaceae*, *Lactobacillus* group, *Enterococcus* spp., *Streptococcus* spp. and *Clostridium* cluster XIV, IV and I) were selected to amplify bacterial groups that were previously reported to be high and low abundant in pig feces [4,5,6,12]. Primer sets and amplification conditions were previously reported [4,12]. Quantification of DNA was performed using Brilliant II SYBR Green QPCR Low ROX master mix (Agilent Technologies), forward and reverse primers (62.5 pmol/µL) and 1 µL (20 ng) of genomic DNA in a final volume of 25 µL on the Stratagene Mx3000P QPCR System (Agilent Technologies, Santa Clara, CA). Amplifications consisted of initial denaturation at 95 °C for 10 min, followed by 40 cycles of 95 °C for 15 s, annealing for 30 s, and elongation at 72 °C for 30 s [4]. Fluorescence was measured at the last step of each cycle. Standards and samples were run on the same plate in duplicate; similarly, negative controls without template DNA were included in duplicate. Melting curve analysis was performed to determine the specificity of the amplification. The dissociation of PCR products were monitored by slow heating with an increment of 0.1 °C/s from 55 to 95 °C, with fluorescence measurement at 0.1 °C intervals. Correct PCR product length was additionally verified by horizontal gel electrophoresis. 

For quantification of bacterial 16S rRNA gene copies, standards were prepared by making serial dilutions (10^7^ to 10^3^ molecules/µL) of the purified and quantified PCR products generated by standard PCR and genomic DNA from pig intestinal digesta [4]. Amplification efficiencies were calculated according to the following equation: E = 10^(−1/slope)^ (E = 1.92 to 1.97). Linear relationships between quantification cycle (Cq) and log of DNA concentration were observed for each primer pair (*R*^2^ = 0.996–0.999). Bacterial abundances were expressed in three different manners, which are commonly used to present bacterial data from qPCR analysis as log_10_ gene copies per gram feces, log_10_ gene copies per nanogram DNA and proportion of total bacterial 16S rRNA gene abundance. Gene copy numbers of total bacteria and target bacterial groups were determined by relating the Cq values to standard curves. The final copy numbers of total bacteria and target bacterial groups per gram feces or ng DNA were calculated by considering the DNA concentration of each sample, the dilution volume and sample weight subjected to DNA extraction [4,12]. Bacterial abundance was also expressed relative to the amplification as proportion of total eubacterial 16S rRNA gene copies, thereby considering the experimentally derived amplification efficiency for each primer set [13]. 

### 2.4. Statistical Analyses

Data were analyzed for normality using the Shapiro-Wilk test. To compare differences between the freezing and storage conditions, data were subjected to ANOVA using the MIXED procedure of SAS (version 9.2; SAS Inst. Inc., Cary, NC, USA) using the pig as the experimental unit and the fixed effects of experiment. Means were reported as least-squares means ± standard error of the mean (SEM) and *p* ≤ 0.05 and 0.05 < *p* < 0.10 were defined as statistically significant and tendency towards significance, respectively. Degrees of freedom were approximated using Kenward-Rogers method (ddfm = kr). Linear discriminate analysis was performed using JMP10 software (SAS Stat Inc., Cary, NC, USA) with the bacterial results as covariates and freeze storage condition as the categorical variable. Linear discriminant analysis results were visualized using the first 2 principal components of the scores plot to identify characteristic trends or grouping among storage conditions of fecal samples.

## 3. Results and Discussion

### 3.1. DNA Yield and Total Bacterial 16S rRNA GENE Abundance 

**Figure 1:**
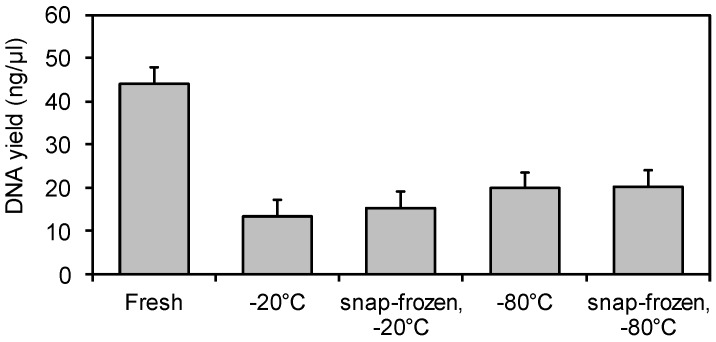
Least square means of DNA extraction yields (ng/µL) from 250 mg fresh fecal samples and freeze-stored fecal samples (−20 °C and −80 °C with or without prior snap-freezing in liquid nitrogen). Polynominal contrasts: DNA extraction from fresh *vs.* frozen feces; *p* < 0.001; storage at −20 °C *vs.* −80 °C, *p* = 0.143; snap-freezing *vs.* none prior to freeze-storage, *p* = 0.761. Bars represent standard error of the mean, *n* = 6.

The DNA yields from pig fecal samples ranged from 7.5 to 64.1 ng/µL in 250 mg feces and were on average 25 to 30 ng higher (*p* < 0.001) after immediate DNA extraction from fresh feces compared to freeze stored feces (Figure 1). In contrast, similar DNA yields were obtained from fresh and corresponding frozen human stool samples [7] using the same DNA isolation kit as in the present study. These opposite findings demonstrate that freeze-storage effects observed for human stool samples may not be readily transferred to fecal samples of pigs. A potential reason may be in relation to the diverging composition in the bacterial communities of the distal large intestine in humans and pigs [10].

Absolute quantification of total bacterial 16S rRNA genes showed a similar impact of freeze-storage on total bacterial abundance as was found for the DNA yield in the present study. The DNA extraction from fresh feces resulted in higher (*p* < 0.001) total bacterial 16S rRNA gene copies per gram feces compared to DNA extraction from freeze stored samples (Table 1). Random shearing and thus fragmentation of DNA during freeze-storage may bias the PCR outcome [14]. Melting curve analysis and horizontal gel electrophoresis, however, indicated that this was not the case in the present study. Snap-freezing in liquid N_2_ and storage temperature did not influence (*p* > 0.10) the DNA yield and total bacterial gene copy numbers per gram fecal sample in the present study. These findings were in accordance with results for human stool which were determined using the same DNA isolation kit and qPCR [7]. 

### 3.2. Bacterial Composition

Differences in the cellular composition of gram-positive and gram-negative bacteria may lead to different extraction or stability of PCR amplifiable DNA from bacteria after freeze storage [7]. For human stool samples, controversial results exist about the effect of freeze storage on the fecal bacterial community. Some studies reported that both relative proportions and absolute abundances of important gut bacterial populations were altered by freeze storage [7,14], whereas others reported little impact of freeze storage on the human fecal bacterial community [15,16]. For instance, an increased Firmicutes to Bacteroidetes 16S rRNA gene ratio was reported for frozen human stool samples compared to fresh samples using qPCR [7]. Likewise, Bacteroidetes, Firmicutes, Actinobacteria and Proteobacteria abundances in stool samples from humans were influenced by storage conditions when samples were analyzed using next-generation sequencing (*i.e.*, 454-sequencing) [14].

In a more recent study using MiSeq sequencing of human stool, in turn, Fouhy *et al.* [15] found little alterations in the fecal bacterial community when freeze storage at −80 °C with or without prior snap-freezing in liquid nitrogen were compared to DNA extraction from fresh samples. In fact, relative bacterial abundances at phyla and family level were not changed by freeze storage and prior snap-freezing [15]. However, at genus level two genera, *Faecalibacterium* and *Leuconostoc* were higher and lower in snap-frozen samples compared to fresh samples, respectively [15]. The controversial results for freeze storage effects on human stool samples may be related to the molecular biological technique used to study the fecal microbiota, the DNA extraction procedure, number of volunteers involved and the host-individual gut bacterial composition [7,14,15,16]. 

**Figure 2:**
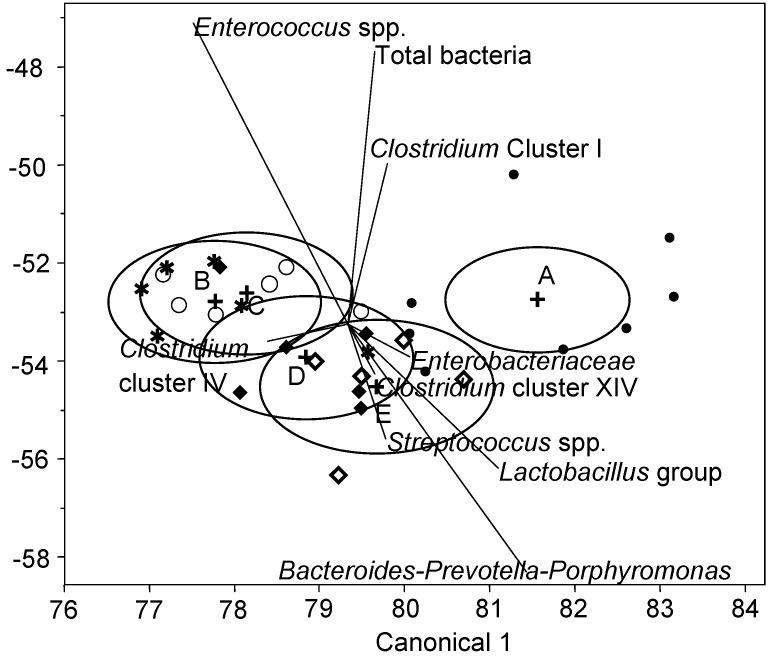
Linear discriminant analysis of the freeze-storage conditions and absolute bacterial abundances in feces. Circles indicate 95% confidence intervals. (**A**) DNA extraction from fresh fecal samples (●); (**B**) from samples stored at −20 °C (○); (**C**) from samples stored at −80 °C (★); (**D**) from snap-frozen samples stored at −20°C (◆); and (**E**) snap-frozen samples stored at −80 °C (◇); *n* = 6.

In the present study, we used a qPCR-based approach as a widely used technique to quantify alterations in the gut microbial community of livestock animals to dietary and medical treatments and disease conditions [4,5,6,17]. Similar to results for human stool samples [7], current qPCR results suggested that the extractability of certain bacterial taxa in feces of pigs may change after freeze-storage as differences in bacterial taxa abundance could be mainly established between fresh and freeze stored fecal samples. Accordingly, the linear discriminant analysis divided the effects into two distinct clusters (Figure 2), clearly separating the bacterial results of fresh fecal samples from those of freeze stored fecal samples. 

In the present study, trends for the influence of freeze storage were mostly similar for the 8 investigated taxa within the *Firmicutes,* *Bacteroidetes* and *Proteobacteria* phyla. However, present observations differed from results reported for human stool samples (e.g., [7,14,15]). Current primer sets were selected to amplify high and low abundant bacterial groups and thus should have covered a representative subset of the bacterial community in pig feces [6,12]. When looking at the absolute abundances of all targeted bacterial groups in one gram feces, those were higher (*p* < 0.05) with DNA extraction from fresh feces compared to freeze stored samples (Table 1). This was likely associated with the higher DNA yield after DNA extraction from fresh feces. When expressing the bacterial data as relative abundance, however, the proportion of *Lactobacillus* group, *Enterococcus* spp., *Streptococcus* spp., *Clostridium* cluster IV, *Bacteroides*-*Prevotella*-*Porphyromonas* and *Enterobacteriaceae* in total bacteria showed the opposite effect by being increased (*p* < 0.05) in freeze stored feces than in fresh feces (Table 1).

The overlapping 95% confidence intervals between the different freeze-storage conditions with or without snap-freezing in the scores plot of the linear discriminant analysis (Figure 2) supported that snap-freezing and storage temperature had little influence on the bacterial composition, thereby being in line with recent observations made for human stool samples using next-generation sequencing [15]. Nevertheless, bacterial composition in feces were greatest apart for freeze storage at −20 °C and storage at −80 °C with prior snap-freezing in liquid nitrogen and bacterial taxa seemed to differently correlate with the various sample processing conditions. The scores plot indicated that *Enterobacteriaceae*, *Clostridium* cluster IV, *Bacteroides-Prevotella-Porphyromonas, Lactobacillus* group and *Streptococcus* spp. discriminated best with snap-freezing prior to freeze-storage, whereas *Clostridium* cluster IV and *Enterococcus* spp. correlated more to simple freeze-storage at −20 °C and −80 °C without prior snap-freezing in liquid nitrogen. Although an increase in gene copies per gram feces was found for *Bacteroides-Prevotella-Porphyromonas* (*p* = 0.033) and similar trends were found for *Clostridium* cluster XIV (*p* = 0.075), and *Enterobacteriaceae* (*p* = 0.060) when stored at −80 °C compared to −20 °C, the dimensions of these changes were 0.1 to 0.2 log units and are therefore not of great physiological relevance. Likewise, the relative abundance of *Enterococcus* spp., when expressed as gene copies/ng DNA, decreased (*p* = 0.032) when feces were stored at −80 °C compared to −20 °C, but changes were also very small. By contrast, snap-freezing reduced (*p* < 0.05) the abundance of *Clostridium* cluster IV when expressed as a percentage of total bacteria. This was particularly the case for samples stored at −80 °C, when prior snap-freezing reduced its abundance by one-third. Effects of freeze storage and snap freezing on the bacterial composition often varied when qPCR results were expressed on absolute or relative terms. Therefore, our data also showed that the way in which bacterial abundances are expressed noticeably modified the effect of freeze-storage on bacterial abundances in pig feces. For instance, when expressing the bacterial abundance as gene copies per nanogram DNA, all bacterial groups except *Clostridium* cluster IV and XIV and *Bacteroides-Prevotella-Porphyromonas* were increased when feces samples were frozen prior to extraction of DNA compared to immediate extraction of DNA from fresh feces. However, when the data were expressed as a percentage of total bacteria, only the *Clostridium* clusters XIV and I of the targeted bacterial taxa were not affected by freeze-storage prior to DNA extraction. 

## 4. Conclusions

Our results demonstrated that freezing of pig feces prior to DNA extraction significantly reduced the resultant DNA yield, the absolute bacterial abundance and modified the bacterial profile when compared to immediate DNA extraction from fresh feces. Moreover, our data further indicated that the findings reported for the freeze storage effect on human stool samples can only be partly transferred to pig fecal samples. Effects of the freezing procedure, *i.e.,* snap-freezing in liquid nitrogen prior to storage, and storage temperature on bacterial abundances were small indicating that bacterial results from qPCR are mostly comparable when different freeze storage conditions were used. However, it should be kept in mind that freeze storage effects differed when bacterial data were presented as relative or absolute abundance. Our present results therefore suggest extracting DNA immediately after sampling of pig feces. Because immediate DNA extraction after sample collection is often not possible, standardization of handling and storage conditions for pig fecal samples is still recommended for comparability of qPCR data within and between studies. 

## Figures and Tables

**Table 1:** Effect of freezing condition on bacterial composition in pig feces **^1^**

Snap-Freezing in Liquid N_2_	No	No	Yes	No	Yes	SEM	Contrasts (*p*-Value)		
Storage Temperature	No	−20 °C	−20 °C	−80 °C	−80 °C		DNA Extraction from Fresh *vs.* Frozen Feces	Storage at −20 °C *vs.* −80 °C	Snap-Freezing *vs.* None Prior to Freeze-Storage
**Log_10_ Gene Copies/G feces**									
Total Bacteria	10.8	10.1	10.1	10.2	10.2	0.09	<0.001	0.176	0.800
*Lactobacillus* group	7.6	7.4	7.4	7.5	7.4	0.35	0.021	0.477	0.892
*Enterococcus* spp.	5.7	5.5	5.6	5.6	5.6	0.34	0.018	0.718	0.771
*Streptococcus* spp.	7.9	7.6	7.6	7.7	7.7	0.28	0.035	0.454	0.685
*Clostridium* cluster XIV	9.3	8.9	9.0	9.0	9.1	0.07	<0.001	0.075	0.257
*Clostridium* cluster IV	8.7	8.24	8.25	8.42	8.27	0.12	<0.001	0.303	0.461
*Clostridium* cluster I	8.0	7.7	7.8	7.8	7.7	0.34	0.022	0.750	0.684
*Bacteroides-Prevotella-Porphyromonas*	10.2	9.7	9.7	9.8	9.8	0.08	<0.001	0.033	0.665
*Enterobacteriaceae*	7.1	6.7	6.9	7.0	6.9	0.60	0.011	0.060	0.516
**Log_10_ gene copies/ng DNA**									
Total Bacteria	6.5	6.3	6.3	6.3	6.3	0.04	<0.001	0.666	0.885
*Lactobacillus* Group	3.4	3.6	3.6	3.6	3.6	0.34	<0.001	0.104	0.945
*Enterococcus* spp.	1.4	1.7	1.7	1.6	1.6	0.34	<0.001	0.032	0.907
*Streptococcus* spp.	3.6	3.8	3.8	3.7	3.7	0.29	0.004	0.241	0.798
*Clostridium* Cluster XIV	5.1	5.2	5.2	5.1	5.2	0.05	0.276	0.583	0.429
*Clostridium* Cluster IV	4.4	4.4	4.4	4.4	4.3	0.09	0.693	0.532	0.103
*Clostridium* cluster I	3.7	3.9	3.9	3.8	3.8	0.35	0.032	0.119	0.827
*Bacteroides-Prevotella-Porphyromonas*	5.9	6.0	5.9	5.9	5.9	0.07	0.636	0.616	0.671
*Enterobacteriaceae*	2.9	3.0	3.1	3.1	3.1	0.59	0.013	0.749	0.532
**Relative Abundance of Bacterial Groups Expressed as Proportion of Total Bacteria (%)**									
*Lactobacillus* Group	0.61	0.96	0.92	0.91	1.02	0.265	<0.011	0.850	0.740
*Enterococcus* spp.	0.007	0.014	0.013	0.013	0.011	0.006	0.041	0.668	0.449
*Streptococcus* spp.	0.34	0.73	0.74	0.68	0.70	0.244	<0.001	0.603	0.869
*Clostridium* Cluster XIV	6.26	7.35	7.97	7.40	8.54	1.239	0.236	0.794	0.460
*Clostridium* Cluster IV	1.09	1.66	1.53	1.81	1.22	0.266	0.007	0.595	0.031
*Clostridium* Cluster I	1.54	1.69	1.61	1.74	1.60	0.894	0.461	0.903	0.500
*Bacteroides-Prevotella-Porphyromonas*	25.77	41.72	42.74	42.41	42.59	7.059	<0.001	0.931	0.848
*Enterobacteriaceae*	0.11	0.26	0.23	0.21	0.18	0.179	0.069	0.335	0.611

^1^ Values are LS means ± SEM, *n* = 6.
